# UK waiting time targets in lung cancer treatment: are they achievable? Results of a prospective tracking study

**DOI:** 10.1186/1749-8090-2-5

**Published:** 2007-01-12

**Authors:** Mohan P Devbhandari, Sing Yang Soon, Pauline Quennell, Philip Barber, Piotr Krysiak, Rajesh Shah, Mark T Jones

**Affiliations:** 1 Department of Cardiothoracic Surgery, Wythenshawe Hospital, Manchester, UK; 2 Department of Clinical Audits, Wythenshawe Hospital, Manchester, UK; 3 Department of Respiratory Medicine, Wythenshawe Hospital, Manchester, UK

## Abstract

**Background:**

Recent guidelines have specified a number of waiting time targets to prevent delay in the treatment of lung cancer. This study was carried out to assess the quality of lung cancer services and compare with national recommendations.

**Methods:**

All newly diagnosed cases of lung cancer presenting to our institution via general practitioner referral were entered into a prospective tracking study by a dedicated audit officer. From September 2003 to March 2005 a total of 247 patients were entered into the study. Of these 133 (54%) were referred by general practitioners and the remainder 114 (46%) were internal referrals. The Cancer Plan waiting time targets are mainly applicable to GP referrals, which formed the study group.

**Results:**

All the patients were seen in chest out-patients clinic within the recommended two weeks period. However there was a delay in starting all forms of treatment. The median waiting time to any form of treatment was 60 days (recommendation 62 days for all patients).

**Conclusion:**

This data demonstrates that although patients receive out patient consultation in the recommended time period, the National Cancer Plan 62 days GP referral to treatment target is not being achieved. A concerted effort by all clinicians is required to meet the prescribed target times.

## Background

The prognosis for lung cancer remains poor with overall five-year survival of 5–10%. This has changed little in the past two decades [1] and is attributed to delays in presentation, diagnosis, staging and treatment. Among the recommendations of the UK (United Kingdom) National Cancer Plan has been the introduction of multi-disciplinary team meetings and an interval of 14 days from urgent GP (general practitioner) referral to first outpatient assessment, and 62 days from GP referral to first mode of treatment [2,3]. Prospective tracking studies was designed with the objective of monitoring the waiting times to treatment for lung cancer at South Manchester University Hospital (SMUHT) and compare it with the national recommendations. SMUHT is a major UK teaching hospital receiving secondary and tertiary referral for thoracic oncology services which has all on site facilities for lung cancer diagnosis and treatment.

## Materials & methods

From September 2003 to August 2004 all suspected primary lung cancer referrals to the chest clinic at our institution were tracked prospectively by a dedicated researcher (Pauline Quennell) to identify patients with newly diagnosed lung cancer. Additional methods were used to capture patients who presented directly to casualty or by internal referral from other departments which included regular interval screenings of histology results, chest radiology reports, International code of diseases codes, thoracic surgery database and Macmillan referrals. Patients presenting from areas outside the primary catchment area were excluded from the study.

At our institution all the referrals are first assessed by respiratory physicians in out patient's clinic or in the ward. Following the diagnostic work up including chest x ray, bronchoscopy, lung function tests, CT scan ± needle biopsy, the patients are discussed in the multi disciplinary team meetings. Increasing number of patients are having PET scans as a part of their investigation. Following discussion at the multi-disciplinary team meeting (MDT) a treatment plan is formulated and appropriate specialist referrals are made. Those patients who need further investigations such as exercise test, angiogram, bone scan, echocardiography etc. to assess the suitability for radical treatment are re-discussed in the MDT in the light of the new results and followed by formulation of treatment plan and appropriate specialist referrals.

The NHS Cancer Plan waiting time targets [2] are mainly applicable to GP referrals, which formed the study group for this paper. Waiting times to treatments were calculated as median days (inter quartile range) in accordance with the National Cancer Plan guideline. Urgent GP referral to date first seen in out patients was calculated by subtracting date of receipt of urgent referral from the date first seen in chest out patients clinic. Similarly urgent GP referral to date of first definitive treatment was calculated by subtracting date of receipt of urgent GP referral from the date of commencement first definitive treatment (any of the three modalities). Chest out patient to surgery intervals was calculated by subtracting date of surgery from the date of out patient consultation. Oncology referral intervals were calculated from the MDT decision for referral to the date of actual start of treatment.

Patients in whom tissue diagnosis was achieved successfully by first Invasive diagnostic method and did not require any extra work up for commencement of treatment were termed simple pathway patients. In contrast to this those patients who required more than one methods or attempts at tissue diagnosis or those who required additional investigations apart from diagnostic and staging work up were deemed to be complex pathway patients. Simple pathway patients who had positive diagnosis obtained at first bronchoscopy were compared with those who had negative bronchoscopy.

## Results

There were 247 new lung cancer patients, of which 133 (54%) were GP referrals and 114(46%) were non-GP referrals. The latter group consisted of 69 casualty and 45 internal referrals. There were 159 male and 88 female patients with median age of 71 years (range 31–89 years).

**Figure 1:**
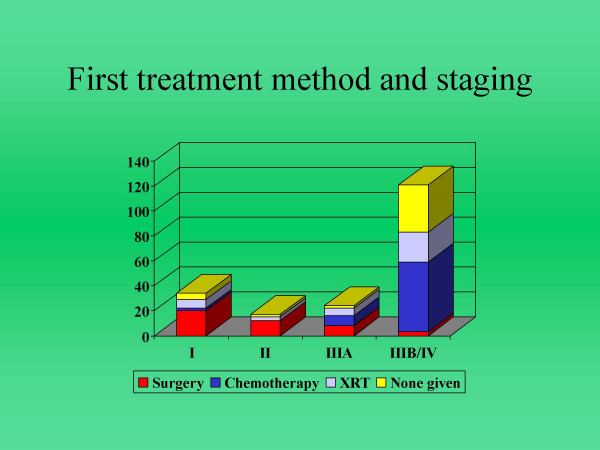
Staging of 188 NSCLC patients and their first methods of treatment.

Histological diagnoses were obtained in 204 (82.6%) patients, which consisted of small cell (SCLC) in 33 (13.3%), non-small cell (NSCLC) in 170 (69%) and mixed in 1 patient (0.004%). TNM staging was available for 188 patients who were clinically considered to be NSCLC including 18 patients without histological confirmation who were treated as NSCLC on clinical grounds alone. The NSCLC patients were staged as I, II, IIIa and IIIb-IV in 13.8%, 7.9%, 12.8% and 65.4% respectively. Formal staging was not applied to 26 unfit patients (Figure 1).

The overall treatment modalities used were surgery, chemotherapy and radiotherapy in 17.4%, 36.8% and 17.4% respectively. The remaining 28.4% did not receive any treatment because of patient choice or poor condition. Out of 33 SCLC patients 24 received chemotherapy and 2 received radiotherapy. Of the remaining 7 SCLC patients, 5 died and 2 declined treatment. Similarly among NSCLC patients 43, 65, and 27 patients received surgery, chemotherapy and radiotherapy respectively while 35 received no treatment (Table 1).

**Table 1:** Histology and treatment methods of lung cancer patients (n = 247).

Histology type	Surgery	Chemotherapy	Radiotherapy	No treatment	Total
Small cell	0	24	2	7	33
Non-small cell	43	65	27	35	170
No histology	0	2	14	28	44
All	43	91	43	70	247

**Figure 2:**
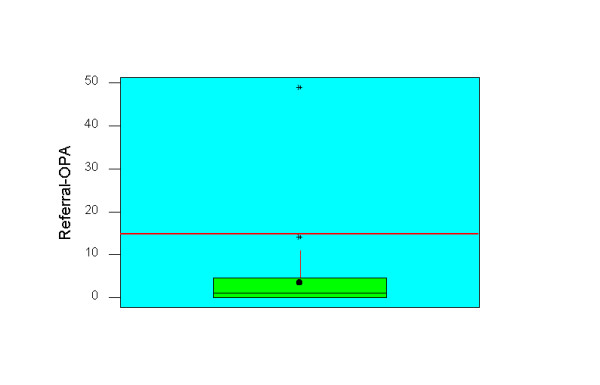
Box and Whisker plot of urgent GP referral to outpatient intervals (n = 133). The red line indicates recommended waiting time and the whisker represents median. The box shows the inter quartile range from 25^th ^to 75^th ^percentile.

**Figure 3:**
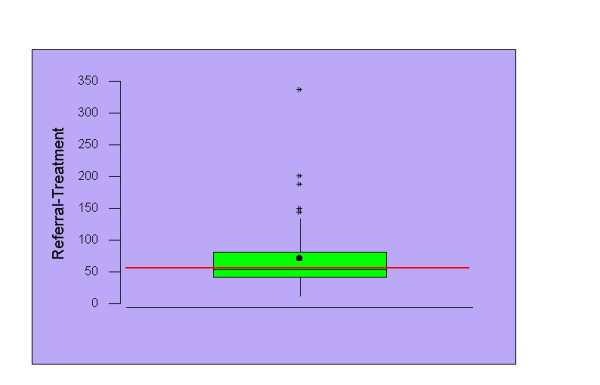
Box and Whisker plot of urgent GP referral to first mode of treatment (n = 133). The red line indicates recommended waiting time and the whisker represents median. The box shows the inter quartile range from 25^th ^to 75^th ^percentile.

Median intervals in days (inter quartile range) for urgent GP referral to chest out patient assessment and first definitive treatment were 1 (0–5) and 60 (44–85) respectively (figures 2 &3). Patients spent a median of 16.5, 25 and 43 days from the time of specialist referral waiting for chemotherapy, surgery and radiotherapy to commence.

Out of 133 GP referral patients 130 (98%) had a bronchoscopy. The first bronchoscopy achieved tissue diagnosis in 64 (49%) patients while it was unsuccessful in 55 (51%). GP referral to treatment intervals for the bronchoscopy positive group was 44 (37–60) days compared to 80 (54–107) days for bronchoscopy negative group which was highly significant (p < 0.01).

## Discussion

The adverse impact of prolonged waiting time on the out come of lung cancer treatment has been well established [4,5]. Delays in lung cancer treatment can be divided into pre-hospital delay and hospital delay. Pre-hospital delay i.e. delay from the onset of symptoms to presentation are largely dependent on severity of symptoms, level of education and complex socio-economic factors that are less within the control of physicians. This can only be improved by addressing wider health issues involving public education, improved awareness and socioeconomic development. There is however scope for improvement in hospital delay i.e. after presentation of the patient to the physician. The published guidelines aim to decrease this phase of delay.

This study emphasizes that previously reported delays [6-8] in treatment of lung cancer persist despite multidisciplinary team meetings and the focus on waiting time targets. Most patients are seen for consultation within the prescribed target of two weeks. The median interval of 60 days between referral and treatment, however, shows that the NHS Cancer Plan target is exceeded in 50% cases. Similar delays have been reported from European countries [7,8] as well as Canada [9].

Most of the delay in our patients is attributable to complex patient pathways and the waits for investigations and initiating treatment. This is most marked in patients who need multiple investigations for diagnosis, staging or assessment of fitness, where each delay or repeat MDT discussion can have a significant cumulative effect [10]. This is clearly evident from comparison of waiting times between the patients who had positive tissue diagnosis at first bronchoscopic biopsy and those whose initial biopsies were negative. This reflected the need for multiple additional investigations in these patients. Only a small proportion of the delay was found to be due to patient factors such as not keeping appointments.

The excessive waiting time occurs for all three modalities of treatment. Limited availability of radiotherapy facilities and consequently a long waiting for treatment is mainly responsible for longest waiting time to treatment in this subgroup. This has been emphasized by previous studies from other centres as well [5,9]. Surgically treated patients underwent greater number of diagnostic and staging procedures including mediastinoscopy as well as additional workup for assessment of fitness for surgery which resulted in longer waiting time. Scarcity of trained thoracic surgeons and limited theatre time [11] are well known. Christie Hospital is the sister hospital specializing in non surgical oncology services, belonging to the same trust (South Manchester University Trust) but located at a short distance away. Some of the patients and their documents had to travel back and forth between the two sites which also contributed to small fraction of the delay.

In addition to its adverse impact on the outcome, delays also cause psychological stress on patients and families [1]. In a recent study 21% of potentially resectable tumours became incurable while waiting on the waiting list and there was an increase in cross-sectional tumour size of up to 373% [5]. This study clearly suggests the current excessive waiting times are not acceptable and needs improving. More concerted effort at integrated multidisciplinary diagnosis and treatment clinics are required. We are in the process of developing guidelines in order to streamline the process of diagnostic workup and assessment for fitness in high risk complex patients. There should also be a significant expansion of infrastructure to meet the prescribed target times.

## Conclusion

In conclusion this data demonstrates that in our sample population, current waiting time targets are still not being achieved in all areas. The majority of patients receive out patient consultation in the recommended time period. Subsequently, however there is an excessive wait for the commencement of all three treatment modalities.
